# A Review of Translational Magnetic Resonance Imaging in Human and Rodent Experimental Models of Small Vessel Disease

**DOI:** 10.1007/s12975-020-00843-8

**Published:** 2020-09-16

**Authors:** Michael S. Stringer, Hedok Lee, Mikko T. Huuskonen, Bradley J. MacIntosh, Rosalind Brown, Axel Montagne, Sarah Atwi, Joel Ramirez, Maurits A. Jansen, Ian Marshall, Sandra E. Black, Berislav V. Zlokovic, Helene Benveniste, Joanna M. Wardlaw

**Affiliations:** 1 Brain Research Imaging Centre, Centre for Clinical Brain Sciences, University of Edinburgh, Edinburgh, UK; 2 UK Dementia Research Institute, Edinburgh Medical School, University of Edinburgh, Edinburgh, UK; 3 Department of Anesthesiology, Yale School of Medicine, Yale University, New Haven, CT USA; 4 Zilkha Neurogenetic Institute, Keck School of Medicine, University of Southern California, Los Angeles, CA USA; 5 Department of Physiology and Neuroscience, Keck School of Medicine, University of Southern California, Los Angeles, CA USA; 6 Heart and Stroke Foundation Canadian Partnership for Stroke Recovery, Sunnybrook Research Institute, University of Toronto, Toronto, ON Canada; 7 Department of Medical Biophysics, University of Toronto, Toronto, ON Canada; 8 Hurvitz Brain Sciences Research Program, Sunnybrook Research Institute, Toronto, ON Canada; 9 Edinburgh Preclinical Imaging, Centre for Cardiovascular Science, University of Edinburgh, Edinburgh, UK; 10 Department of Medicine (Neurology), University of Toronto, Toronto, ON Canada

**Keywords:** Small vessel disease, Magnetic resonance imaging, Systematic reviews, Lacunar infarcts, Dementia, Brain imaging

## Abstract

Cerebral small vessel disease (SVD) is a major health burden, yet the pathophysiology remains poorly understood with no effective treatment. Since much of SVD develops silently and insidiously, non-invasive neuroimaging such as MRI is fundamental to detecting and understanding SVD in humans. Several relevant SVD rodent models are established for which MRI can monitor in vivo changes over time prior to histological examination. Here, we critically review the MRI methods pertaining to salient rodent models and evaluate synergies with human SVD MRI methods. We found few relevant publications, but argue there is considerable scope for greater use of MRI in rodent models, and opportunities for harmonisation of the rodent-human methods to increase the translational potential of models to understand SVD in humans. We summarise current MR techniques used in SVD research, provide recommendations and examples and highlight practicalities for use of MRI SVD imaging protocols in pre-selected, relevant rodent models.

**Electronic supplementary material:**

The online version of this article (10.1007/s12975-020-00843-8) contains supplementary material, which is available to authorised users.

## Introduction

Cerebral small vessel disease (SVD) is estimated to cause 20–25% of strokes globally and 45–65% of dementias [1, 2]. Magnetic resonance imaging (MRI) is used extensively in clinics and research to identify SVD-associated lesions and imaging biomarkers. Key SVD-related features, image acquisition and quantification methods are summarised in recent position papers [3, 4].

The core diagnostic SVD protocol includes the following: T1-weighted (T1-w), to provide detailed anatomical images, identify brain atrophy and differentiate grey/white matter; T2-weighted (T2-w), to distinguish lacunes from dilated perivascular spaces (PVS) or white matter hyperintensities (WMH); fluid-attenuated inversion recovery (FLAIR), to identify WMH, lacunes and established infarcts; diffusion-weighted imaging (DWI), to detect recent small infarcts due to high sensitivity to acute ischaemia [5]; and blood sensitive (gradient echo (GRE)/T2*-w or susceptibility-weighted imaging), to identify cerebral microbleeds, superficial siderosis and mineral deposition [3].

Advanced MRI techniques aid research into SVD pathogenesis [6] including DTI, to assess white matter integrity, and methods, to assess microvascular function, including cerebrovascular reactivity (CVR), intracranial vascular and cerebrospinal fluid (CSF) pulsatility, cerebral blood flow (CBF) and blood–brain barrier (BBB) integrity. Dynamic and static CBF is important in assessing cerebrovascular health [7]. Dynamic blood flow responsiveness is commonly assessed with blood oxygenation level–dependent (BOLD) [8]. Resting CBF can be measured by dynamic susceptibility contrast (DSC), arterial spin labelling (ASL) and phase contrast MRI (PC-MRI). Dynamic contrast-enhanced (DCE) MRI using intravenous injection of gadolinium (Gd) contrast [9] shows increased BBB permeability in SVD [4, 10–12]. Magnetic resonance spectroscopy (MRS) detects neuro-metabolite changes, including evidence of axonal loss/disruption [13, 14].

Various rodent models reflect different putative SVD mechanisms and some features of human disease [15–19]. Hypertension models replicate elements of microvessel remodelling from some sporadic human SVD [20], including venous collagenosis [21], but hypertension is only one risk factor. The spontaneously hypertensive stroke prone rat (SHRSP) has endothelial dysfunction, microglial, white matter and BBB abnormalities prior to hypertension and sporadic SVD features when older [18]. Bilateral carotid artery microcoils to induce mild stenosis (BCAS) leads to some SVD characteristics [22, 23] but may work through altering carotid elasticity and arterial pulsatility. There are also several monogenic SVD and knockout models [19, 24, 25].

**Fig. 1:**
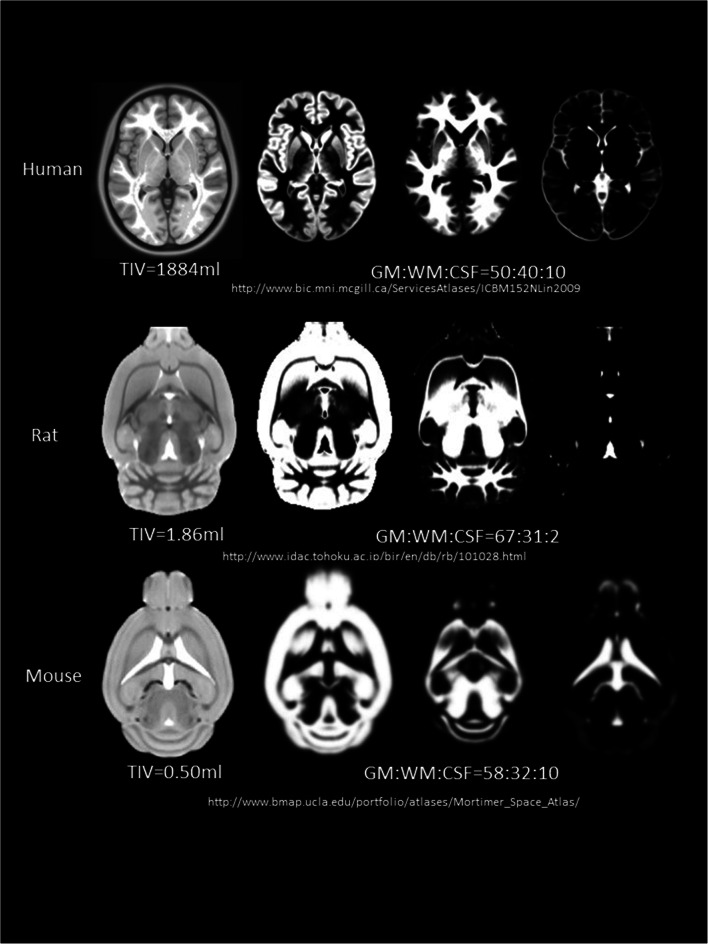
Approximate total intracranial volume (TIV) volume (ml) and grey matter:white matter:CSF ratio in healthy (young) animals shown relative to the human brain based on publicly available templates

There are some inherent limitations to the translational potential of animal models [15, 16, 18]. Anatomically, white:grey matter ratios and brain sizes differ markedly (Fig. 1) [15, 16]; arterial anatomy [26, 27], density, spacing and positioning of penetrating arterioles and draining venules also vary [28]. In stroke models, assessing rodent neurological deficits is challenging when symptoms are mild and recovery may be rapid [29]. Anaesthetics, necessary for many types of study, can affect cerebral haemodynamics and CSF transport [30] and may provide neuroprotective or adversive effects that could alter the tissue changes [31, 32]. Rodent respiratory and heart rates are higher, restricting options for physiological measurements and pulse gating required for some MR sequences at comparable temporal resolution to humans.

Despite the anatomical and physiological differences from humans, rodent SVD models are however key for investigating pathological processes and time trajectories of disease evolution and developing and testing novel therapies [33]. Preclinical MRI facilitates independent validation with contemporaneous histology or other imaging techniques and improves clinical translation and exploration of physiological processes, e.g. fluid flow through the glymphatic system [34–36], glymphatic system changes during sleep [37] and effects of risk factors, including hypertension and diabetes, on tissue damage and microvascular fluid dynamics [38, 39].

As part of efforts to improve the translational value of preclinical models to human SVD, particularly through the use of MRI, we reviewed the literature to identify studies which had adapted clinical MRI methods to preclinical MRI, or vice versa. Our intention is not to review preclinical SVD rodent models but rather to evaluate synergies, strengths and limitations between human and rodent MRI to optimise the translational potential of MRI for non-invasive longitudinal assessment of disease development and progression in SVD.

## Methods

### Systematic Review

We reviewed the literature to extract information on approaches to improving comparability or complementarity of brain MRI techniques between studies in rodents and humans. The systematic literature search was conducted on Medline and Embase from 1946 until April 2020 through Ovid. Exploded headings and search terms relating to SVD were combined with terms for MRI and relevant advanced MR techniques. We also combined these results with a comprehensive search strategy for rodents based on Hooijmans et al.’s filter [40] and terms associated with translational research (e.g. translat*, retranslat*) prior to limiting to papers relating to humans. Finally, we removed duplicates. For the full search strategy, see the Supplementary Material.

We also manually checked reference lists in reviews and original papers for additional relevant references. Other papers were identified from the authors’ libraries. We inspected all identified papers to ascertain whether they satisfied the eligibility criteria. We included papers that provided information on MRI methods designed to be used in humans and those designed for use in rodent models but that aimed to capture SVD features, including static [41] and dynamic biomarkers (e.g. vascular reactivity and BBB dynamics), which use similar sequences in rodents and humans including practical guidance. We excluded studies published solely as conference abstracts due to providing insufficient detail.

One author (MSS) extracted summary data for each paper including the type of publication, diseases covered, imaging techniques employed and a short summary of the focus. Other authors resolved uncertainties. We sought to provide an overview of all published strategies for comparable human-rodent MR imaging protocols, practicalities and advice on specific sequence(s) including structural, post-mortem and dynamic vascular function assessments including CVR and DCE.

## Results

**Fig. 2:**
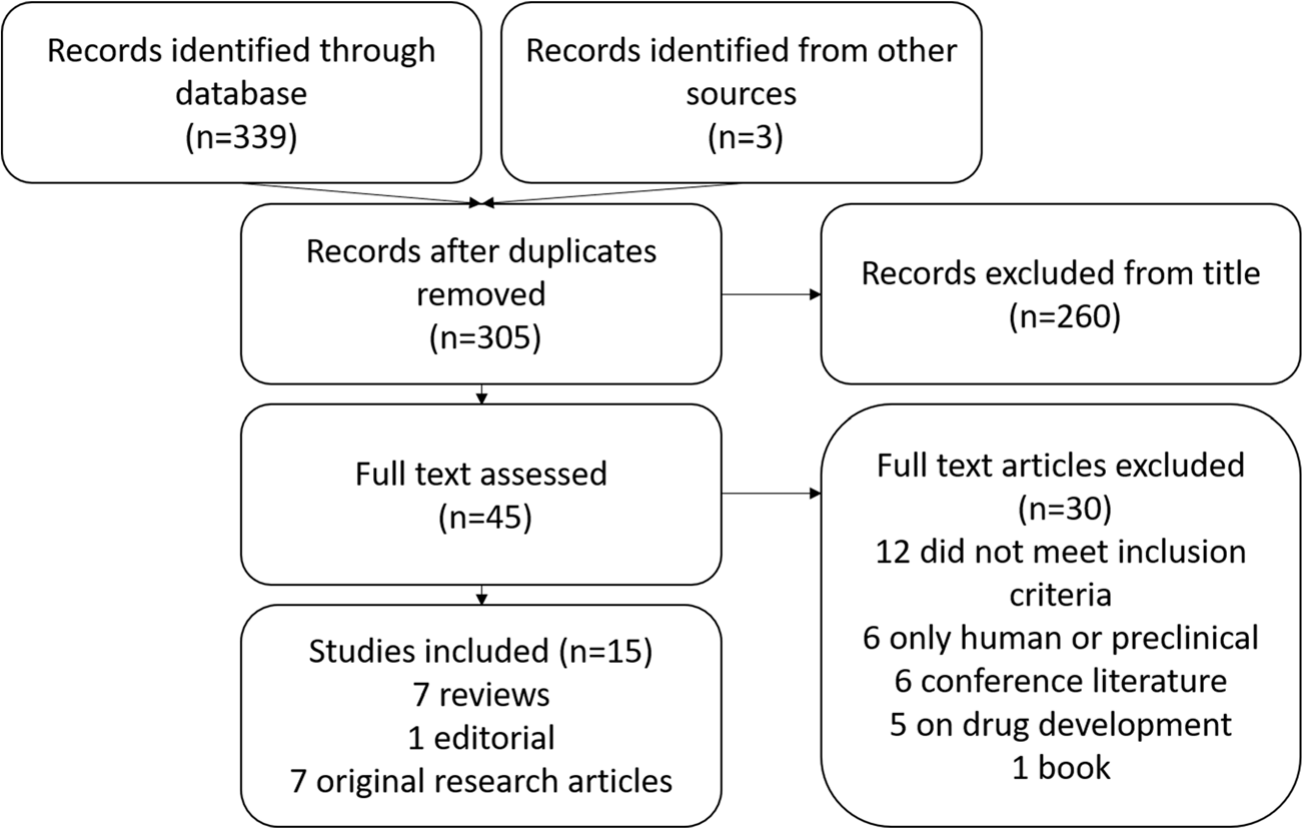
PRISMA flow diagram of the literature search

The search identified 305 unique publications of which we excluded 260 as irrelevant based on the title, mainly due to being in an unrelated population, case reports or modality (e.g. positron emission tomography (PET), computed tomography (CT)) (Fig. 2). On full-text review of the remaining 45, a further 30 were excluded, mainly due to the following: no MRI (12), translation of other biomarkers for drug development (five), clinical or preclinical studies only (six), conference abstracts (six) and one book.

Of the 15 relevant papers that addressed any aspect of rodent-human MRI, seven were narrative reviews [42–48], one was an editorial [49] and seven were original research [22, 50–55] (Table 1). Of these, six focused on AD (four reviews [42–45], one editorial [49] and one original paper [52]), one review [47] on stroke, two original papers on Huntington’s disease [51, 54], one original paper each on ageing [50] and hypertension [55], two original papers [22, 53] and one review on SVD [48] and one review briefly summarised functional MRI (fMRI) applications in several diseases [46], including stroke and neurodegeneration.

**Table 1:** Summary of studies included in the literature review

Authors	Type	Disease	Methods	Focus
Beckmann (2011) [43]	Review	AD	Dynamic susceptibility contrast, arterial spin labelling, MR angiography (MRA) and wall shear stress	Different approaches to measuring cerebrovascular changes in humans and transgenic mice
Braakman et al. (2009) [44]	Review	AD	Structural MRI	Reviews the challenges of imaging amyloid plaques in vivo in mice with MR, building on initial work with ex vivo human brain tissue [58], and summarises the outstanding technical barriers to translating such methods to humans
Delatour et al. (2010) [45]	Review	AD	Structural MRI, diffusion MR, MRA, cerebral blood volume, MRS, PET, microscopy techniques	Overview of imaging biomarkers in AD mouse models and their potential for translation with some consideration of the technical barriers.
Kincses et al. (2015) [42]	Review	AD	Structural MRI (voxel-based morphometry and diffusion-weighted) and molecular imaging	Primarily structural biomarkers including features where findings in human and animal models overlap, challenges of finding animal models mirroring imaging characteristics of the disease in humans
Keene et al. (2016) [49]	Editorial	AD	Structural and functional MRI/PET, CSF and cognitive assessment	Standardised approach to characterising mouse models based on neuropathological features incorporated in guidelines from the National Institute on Aging and Alzheimer’s Association
Meadowcroft et al. (2009) [52]	Original research	AD	Structural MR, histology and electron microscopy	Post-mortem MR, electron microscopy and histology of beta-amyloid plaques in humans with AD and *APP/PS1* transgenic mice to reveal the interplay between the plaques and relaxation mechanisms
Pan et al. (2015) [46]	Review	Stroke, epilepsy, neurodegenerative disease, stress and depression	BOLD MRI	Challenges of translating resting state functional MRI paradigms from humans to rodent models. Limited summary of applications in several different conditions.
Muir et al. (2016) [47]	Review	Stroke	MR/CT angiography and perfusion	Application of imaging as a part of trial recruitment and a study endpoint which can lead to more efficient study design while providing non-invasive measures at an earlier stage
Chaumeil et al. (2012) [51]	Original research	Huntington’s	MR spectroscopy	Rodent-human ^31^P MRS protocol which provides some guidance on the different considerations involved in the two aspects of the study
Sawiak et al. (2013) [54]	Original research	Huntington’s	Structural MRI	Development of voxel-based morphometry for mouse models assessed in a Huntington’s model
Holland et al. (2011) [50]	Original research	Ageing	Structural MRI, DTI and magnetisation transfer (MT)	Translation of DTI and MT protocols to a bilateral common carotid stenosis mouse model of ageing.
Holland et al. (2015) [22]	Original research	SVD	Structural MRI, DTI and histology	Application of radiological assessment and DTI in a bilateral common carotid stenosis mouse model to explore gliovascular alterations
Wardlaw et al. (2020) [48]	Review	SVD	Structural MRI, DCE-MRI phase contrast, and advanced dynamic MRI scans	Review of current knowledge and outstanding questions on PVS function with a focus on the scope for translation
Li et al. (2015) [55]	Original research	Hypertension	Structural MRI, ASL and magnetic resonance angiography	Application of multimodal MRI to assess vessel diameter of cerebral and downstream arteries in relation to the effects on CBF and vascular reactivity
Wang et al. (2020) [53]	Original research	Small vessel diseases	Structural MRI (susceptibility-weighted imaging, SWI)	Use of ultra-small super-paramagnetic iron oxide contrast agents for imaging of small vessels in rodents and humans, with a preclinical histological validation

All studies were cross-sectional [22, 50, 52–55] with the exception of the preclinical component of [51] which was longitudinal.

We found no protocol with guidance on designing longitudinal MRI studies in rodents to mirror typical MRI research to examine disease development in human cohorts. One rodent protocol used DTI, T2w, T1w and T2*w but not FLAIR [22]. No studies addressed image analysis issues that are commonly encountered in human studies, such as image registration and lesion tracking over time, or combining data from several different sequences from the same anatomical regions or lesions at one time point. We did find a few studies where assessment tools developed to assess SVD features in humans, such as the Fazekas scale for WMH [56], Brain Observer MicroBleed Scale (BOMBS) for cerebral microbleeds [57] and several image analysis methods such as segmentation and voxel-based morphometry, have been adapted for use in animal models [22, 54]. We also found one example of a novel imaging approach developed to assess perivascular space fluid uptake in rodents that had been translated to human use [53].

There were few studies where MRI had been used to assess response to treatment in rodents [42, 43]. The review papers highlighted several complementary methods including microscopy [45] and functional [43, 45, 46] and in vivo and ex vivo structural MR imaging [22, 42, 44, 45, 50] (Table 1). However, these methods were only rarely used together in the same study for validation [49]. Lastly, several technology [44] or transferability [45, 46] limitations for clinical to preclinical and vice versa were highlighted, including the need for further validation and methodological advancements to provide scans with higher sensitivity and specificity. There was no detailed overview of a range of MR imaging techniques applied in rodents in disease- or lesion-specific contexts to mirror those developed to study human SVD [3].

## Proposed Approaches to Improve the Potential of Rodent-Human Translational MRI

### Structural MRI in Rodents and Humans

Human SVD features are present in many models [15, 17, 18, 50]. Certain features are less commonly reported; however, PVS were only recently identified clinically and appear on histology in pericyte-deficient mice [19]. Therefore, optimised parameters enhancing visibility of disease-related features should be used and standardised where possible (https://harness-neuroimaging.org/) [4].

**Fig. 3:**
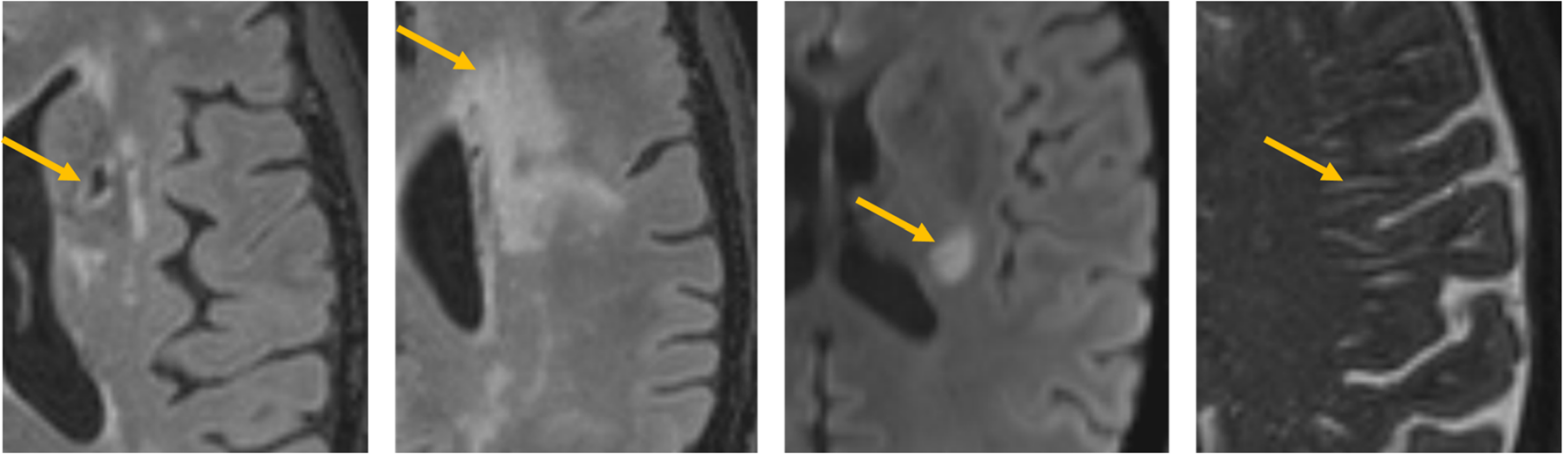
Examples of the appearance of SVD features in human, from left to right: recent small subcortical (i.e. acute lacunar) infarct (DWI), WMH (FLAIR), lacune (FLAIR) and enlarged perivascular spaces (T2-w) indicated by yellow arrows

**Fig. 4:**
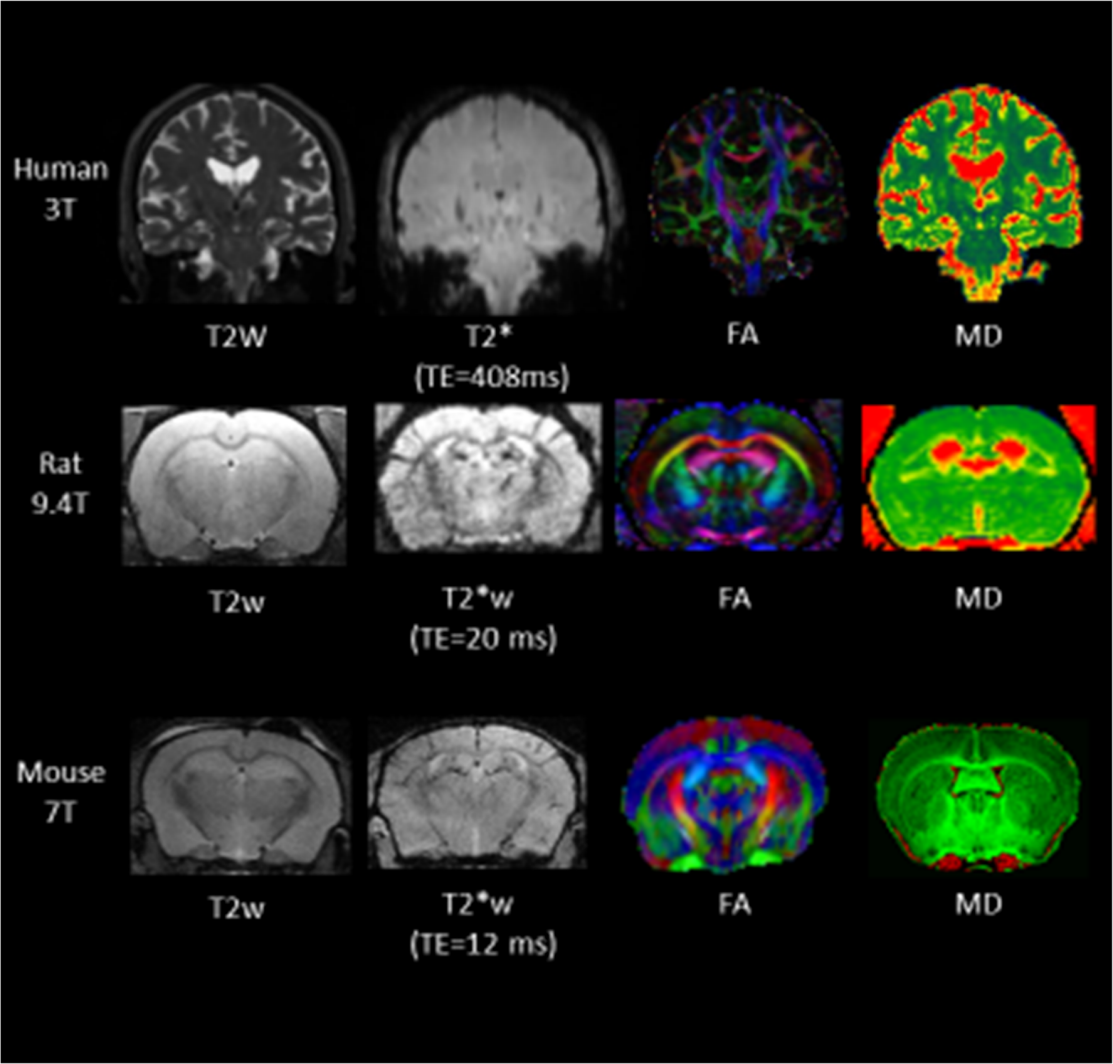
Illustrations of commonly used structural imaging contrasts in human, rat and mouse models (Human 3 T: MS/JMW, Rat: HL, Mouse: AM). 7 T, 9.4 T and even higher field strengths are commonly used in rodent imaging

Translational SVD research must account for practical differences. Rodent imaging needs ultra-high magnetic field strengths (i.e. ≥ 7 T) as a necessary and common means of increasing signal-to-noise ratio due to smaller spatial resolution. In human imaging, acquisitions can be accelerated via several techniques, including compressed sensing and parallel imaging [59, 60], thereby reducing motion artefact while allowing incredibly detailed 3D acquisitions in clinically acceptable times; however, such acceleration methods are often less readily available on preclinical platforms. Variations in preclinical pulse sequences may affect comparability of contrast-to-noise ratio (CNR) and image interpretation (Fig. 3). Applying clinical imaging protocols may better integrate translational work, track disease progression/assessment (Fig. 4) and improve cross-model methodological translatability.

Possible exemplar sequences, based on ones found to be reliable and informative at the authors’ sites, are listed in Table 2. Options include 2D or 3D sequences, but while 3D are desirable, they take longer than 2D, so 2D may be preferable in some situations.

**Table 2:** Exemplar image acquisition parameters for a standard clinical neurovascular structural protocol and possible rodent counterparts at co-authors’ sites

Sequences	Purpose	Typical clinical parameters (3 T)	Typical preclinical parameters for rats (7 and 9.4 T)	Typical preclinical parameters for mice (7 and 9.4 T)
T1w	Detailed anatomy	3D, voxel size: 1 mm isotropic, TR/TE = 2500/4.37 ms, TI = 1100 ms, ~ 4 min	2D, voxel size: 0.20 × 0.20 × 0.8 mm, TR/TE 780/9.5 ms, ~ 5 min	2D, voxel size: 0.07 × 0.07 × 0.4 mm, TR/TE = 700/11 ms, ~ 10 min
T2w	Lacunes, PVS	3D, voxel size: 0.9 mm isotropic, TR/TE = 3200/408 ms, ~ 4 min	2D, voxel size: 0.1 × 0.07 × 0.3 mm, TR/TE = 4000/26 ms, ~ 9 min	2D, voxel size: 0.07 × 0.07 × 0.4 mm, TR/TE = 5000/45 ms, ~ 9 min
FLAIR	WMH, juxtacortical or ventricular lesions	3D, voxel size: 1 mm isotropic, TR/TE = 5000/388 ms TI = 1800 ms, ~ 6 min	2D, voxel size: 0.34 × 0.34 × 1.0 mm, TR/TE 8000/30 ms, TI 1930 ms, ~ 10 min	2D, voxel size: 0.13 × 0.13 × 0.5 mm, TR/TE = 6000/15 ms, TI = 1700 ms, ~ 12 min
T2*w/GRE or SWI	Microbleeds, prior ICH, cortical siderosis, tissue mineral deposition; separate intracranial contents from skull	2D, voxel size: 0.6 × 0.6 × 3 mm, TR/TE = 28/20 ms, flip angle = 9°, ~ 4 min (SWI)	3D, voxel size: 0.08 × 0.08 × 0.03 mm, TR/TE = 32/15 ms, flip angle = 12°, ~ 10 min (GRE)	2D, voxel size: 0.06 × 0.06 × 0.5 mm, TR/TE = 415/5 ms, flip angle = 12°, ~ 10 min (GRE)
DWI/DTI	Acute ischaemic lesions; fractional anisotropy (FA), mean diffusivity, tract integrity, tractography	2D, voxel size: 1.3 × 1.3 × 3 mm, TR/TE = 4000/75 ms, 30 directions, ~ 6 min	2D, voxel size: 0.17 × 0.17 × 0.7 mm, TR/TE = 5000/20 ms, 30 directions, ~ 20 min	2D, voxel size: 0.15 × 0.23 × 0.5 mm TR/TE = 3000/32 ms, 30 directions, ~ 10 min
PD	Separate intracranial contents from skull to measure ICV; a few centres use for lesion differentiation (not used in STRIVE)	3D, voxel size: 1 × 1 × 1 mm, TR/TE = 6.04/2.44 ms, ~ 2 min	2D, voxel size: 0.1 × 0.07 × 0.3 mm, TR/TE = 4000/4 ms, ~ 9 min	2D, voxel size: 0.07 × 0.07 × 0.4 mm, TR/TE = 5000/8 ms, ~ 9 min

Times refer to total sequence acquisition time. *TR* repetition time, *TE* echo time, *TI* inversion time

Validation of pathogenic mechanisms, tissue changes and evolution, complemented by invasive measurements, e.g. 2-photon [38] and post-mortem microscopy, are main reasons for using preclinical models. Preclinical imaging allows longer/more regular scanning and examination using multiple modalities. Advanced structural scans provide useful additional metrics, e.g. DTI for white matter tract integrity/visualisation, and network connectivity assessment, which may help explore cognitive/functional deficits.

Anatomical factors that may affect the translational potential include appropriate metrics/methods to control for relative brain size/tissue ratios and the effects of interventions to increase SVD burden. Clinical SVD features typically increase with age; hence, while using naturally aged rodents [61] may be more challenging and costly, they may reveal more obvious features plus be relevant to longitudinal studies and valuable for preclinical drug testing.

### A Greater Role for Post-Mortem MRI in Humans and Rodents

Post-mortem MR (PM-MRI) shows the neuropathology and macroscopic tissue damage underlying MRI features [62, 63]. Long scans are much more feasible PM yielding better image quality with corresponding benefits to signal-to-noise ratio (SNR), spatial resolution and reduced partial volume, thereby enabling precise MR-histology comparisons. Formalin-perfusion fixation improves contrast-to-noise (CNR) for MR microscopy; while SVD lesions remain conspicuous [51, 64–66], prolonged fixation may also induce signal artefacts obscuring lesions [67] and discriminating between pre- and post-mortem damage on MRI may be more challenging (e.g. small parenchymal haemorrhage versus post-mortem intravascular thrombus) [68]. While perfusion fixation is the gold standard for preclinical models, immersion is often preferred in humans for practical reasons. Optimal perfusion fixation is also important to avoid post-mortem intravascular thrombus mimicking pre-mortem intravascular thrombus for example [69]. The time of sacrifice and fixation method must therefore be carefully considered. There are validated protocols relating pre-mortem and PM human MR-visible SVD lesions to histology [62, 70, 71]. While some features may be less evident at PM, PM-MRI is well-suited to automated analysis and is underused in SVD research.

Few papers directly compare PM-MRI to histology [72–75]. Non-quantitative analyses, assessing overall distribution and size [76], risk missing heterogeneous WMH features, including pathological variation [77]. There are limited data on lesion development in rodents during normal ageing; optimisation studies could explore histological-MRI correlates across the lifespan. Close scrutiny of structural and quantitative images by experts in human MRI may identify lesion development stages on PM-MRI. Ex vivo DTI gives data for large areas of tissue and thus can complement and guide the ‘spot sampling’ approaches typical of histological assessment of white matter integrity [78, 79].

Cerebral microinfarcts (CMIs), small ischaemic lesions encompassing neuronal loss, gliosis and cavitation [80], can appear acutely on diffusion imaging. However, size and signal transience may lead to underestimation of frequency and involvement [71, 81]. PM-MRI allows direct histopathological validation of specific imaging markers [82] including in rodent models. Studies comparing ex vivo MR with histology of CMIs are sparse. More studies would help determine the role of CMIs in SVD and neurodegeneration and help improve the spatial resolution.

Cerebral microbleeds (CMBs), small chronic haemorrhages, appear hypointense on T2*-w/SWI [3, 83] and are not visible on other sequences. CMBs remain visible post-mortem, mirroring histopathology and pre-mortem appearances [84, 85]. Underlying pathological features occurring within CMBs include acute/old (macro)haemorrhages and residual haemosiderin [86]. Paramagnetic properties cause blooming, lesion size overestimation and potential false positives on MRI [87], reinforcing the value of targeted histology alongside MRI measurements. Haemosiderin deposition is detected [19, 88] in amyloid pathology mice, in whom T2*-w CMBs are also reported to correlate with histology [89, 90]. Susceptibility-weighted or quantitative susceptibility imaging may improve sensitivity and accuracy for CMBs, separating haemorrhage from mineralisation, but confirmatory neuropathological studies are warranted [91, 92].

### Advanced Dynamic MRI Methods In Vivo in Rodents and Humans

#### Cerebral Blood Flow and Cerebrovascular Reactivity

CVR refers to vasodilation and vasoconstriction of cerebral microvessels, typically to vasoactive stimuli (e.g. increased CO_2_ inspiration or intravenous acetazolamide). CVR is a quintessential cerebrovascular health measure that reflects the role of blood vessels in regulating CBF, oxygen and nutrient delivery, waste product clearance and dissipating heat [93]. CBF is generally measured at rest, with ASL being a widely used method [94].

Several studies have imaged CBF and CVR in SHR and Wistar Kyoto (WKY) rats [55, 95]. However, anaesthetic agents can affect resting CBF and haemodynamic responses and therefore are particularly important to consider when planning experiments on CBF or CVR. For example, CBF was higher in SHR versus in WKY under 2% isoflurane but not with alpha-chloralose and isoflurane reduced CVR [96]. As in preclinical functional neuroimaging, sedation rather than full anaesthesia is often preferred [46]. Dexmedetomidine depresses global CBF; therefore, in some experiments, general anaesthesia with inhalational agents may be preferable. Anaesthetic protocols should also minimise impact on signal and derived imaging variables; further optimisation studies and standardised reporting of anaesthetic protocols would be beneficial.

CVR requires a physiological manipulation/challenge but voluntary breath holding is unsuited to preclinical studies [97] and controlled hypercapnia via breathing apparatus is more reliable. In rats and larger murine models, intubation provides greatest control of inhaled and exhaled gases and is optimal for preclinical CVR. Sealed chambers are practical where intubation is unviable or lower level sedation is preferred. Since CO_2_ tolerance varies between species [98, 99], tolerability should be balanced against inducing robust signal changes. Humans tolerate a 6% CO_2_ stimulus well [99].

CVR analyses typically use regression models but must account for physiological and practical factors, notably haemodynamic delays, and filtering band frequency may vary under anaesthesia [46].

CVR has not been fully exploited in rodents to better understand how impaired vasoreactivity develops at whole brain level and leads to brain damage in SVD. Longitudinal CVR measurements coupled with multiphoton microscopy via cranial windows or isolated vessel preparations [100] could strengthen the direct validation of in vivo CVR and improve its use as a biomarker and an intermediary outcome in trials of therapeutic interventions.

#### Blood–Brain Barrier Impairment

The BBB plays a central role in brain homeostasis, controlling exchange of fluids and selected molecules while protecting parenchyma from potentially toxic plasma components [101]. BBB breakdown occurs in neurodegenerative diseases, including Alzheimer’s, Huntington’s and SVD [102–107].

MRI, contrast-enhanced CT (CE-CT), PET and biofluid biomarker approaches can measure BBB permeability [4]. MRI methods include dynamic or static contrast-enhanced MRI [4, 11] and non-contrast-based methods (e.g. ASL [108, 109] and T1-w black-blood imaging [110]). The most widely used method to detect subtle focal BBB permeability increases is DCE-MRI [102]. Preclinical validation is limited, but in vivo BBB measures would complement histologic assessment and validation of BBB dysfunction.

DCE-MRI involves T1-mapping followed by intravenous Gd injection and repeated T1-w sequences [111]. Multi-slice or volume sequences, usually GRE or fast low-angle shot, run repeatedly for ca. 20 min; longer acquisitions may be appropriate for low-level leakage [112, 113]. Sequence optimisation balances coverage, SNR and spatial and temporal resolution. As pharmacokinetic analysis requires reliable arterial or venous input functions, identifiable vessels must be covered, e.g. internal carotid arteries or sagittal sinus. High temporal resolution is critical for bolus injections due to rapid blood signal changes [113, 114]. A dilute contrast phantom adjacent to the animal’s head can allow signal-to-concentration transformation correction. Anaesthesia level, temperature and respiration should be monitored and adjusted to minimise input function variation.

BBB function parameters, such as volume transfer constants between extracellular space and plasma, are derived by modelling the Gd concentration-time curve, along with CBF and cerebral blood volume. There is now dedicated software for clinical and preclinical DCE analyses (e.g. *ROCKETSHIP* [115]). Patlak models are best suited to subtle BBB leakage in SVD [112, 113]. Retinal imaging, MR venography [116] and phase contrast MRI [117] have been used as surrogates to direct measurement of vessel diameter in response to stimuli; further studies, particularly in humans at field strengths ≥ 7 T, may provide more detailed insight into properties of the microvasculature. Preclinically, methods like multiphoton microscopy can also complement DCE-MRI by determining the microvascular changes underpinning BBB leakage [118].

There are novel MRI methods in development that use endogenous contrast which may improve sensitivity. For example, DW-pCASL employs diffusion weighting to distinguish labelled blood in the microvasculature from that in brain tissue to measure water exchange [108]. WEPCAST also employs an ASL approach using velocity encoding to isolate venous signal [109]. However, these approaches are hypothesis-based and their translation to humans would be greatly facilitated by first demonstrating that they provide reliable measures of BBB function in preclinical models.

#### MR Spectroscopy to Assess Metabolites

Magnetic resonance spectroscopy (MRS) determines tissue metabolite levels in vivo and would provide a promising approach to explore SVD and neurodegenerative disease progression [14, 119–122]. However, MRS has not been applied extensively in human SVD or preclinical models.

There are some practical limitations. For example, for sampling homogeneous tissue, single voxel spectroscopy (SVS) in mice requires typical volumes ca. 0.008 cm^3^ [123] (0.4% of brain volume) relative to 4 cm^3^ (0.03%) in humans [124] and positioning the sample volume requires structural imaging. Multi-voxel MR spectroscopic imaging (MRSI) [125] increases brain coverage and can examine metabolite distribution, although with longer acquisition times. It is important to control for disease burden and relative proportions of healthy/diseased tissue, particularly in advanced disease, and where atrophy reduces the amount of tissue to sample. It is important to establish the test-retest reliability on individual MRI scanners, particularly for lactate and coupled metabolites [126] before use in experiments although the reproducibility for detecting more abundant metabolites is generally good and several quantification approaches are available [124].

Surface and/or refined coil designs improve rodent MRS sensitivity [127] and higher field strengths provide better spectral resolution and distinction of metabolite peaks. Preclinical MRS allows cross-validation of metabolite concentrations using chemical methods, providing greater confidence scanning humans longitudinally.

Beyond proton MRS, carbon-13, oxygen-17, sodium-23 and phosphorus-31 MRS may be applied, though multi-nuclear equipment is needed. Contrast agents, notably deuterium [128] and thulium [129], can monitor metabolism, pH and temperature in vivo to assess disease progression or changes in ketogenic states.

#### Novel MRI Methods in Rodents and Scope for Rodent-Human Translation

PVS are small conduits that envelop penetrating cerebral arterioles/venules where CSF can exchange with interstitial fluid (ISF) [48]. As part of the glymphatic system, PVS are thought to clear brain fluid and waste, facilitated by aquaporin-4 (AQP4) water channel–mediated CSF-ISF exchange at the peri-capillary space before clearance to lymphatic vessels [34, 130, 131]. While aspects of the physiology are controversial [132, 133], CSF-ISF exchange studies provide opportunities to understand PVS in vascular and neurodegenerative diseases. PVS become enlarged and more visible in SVD and are associated with inflammation, impaired CVR, increased BBB permeability and vascular pulsatility [48]. Their small size makes them difficult to assess in humans. However, CSF delivery to PVS can be characterised in rodents using DCE-MRI and Gd injection into the cisterna magna CSF. Small volume infusion of Gd into the CSF pool during 3D MRI demonstrates spatially and temporally resolved solute transport through the brain [134] and can show altered PVS function with vascular risk factors. For example, type 2 diabetes mellitus rats exhibit slower Gd clearance, with accumulation and retention, enhanced perivascular arterial influx and increased hippocampal signal intensity [39]. Tracer transport mechanisms are highly complex, but pharmacokinetic models [135, 136] and mass transport algorithms [137, 138] help quantify influx/efflux contributions. An optimal mass transport analysis in SHRSP rats reveals reduced and slowed solute transport from CSF into the brain [139]. Though rare, opportunistic human studies show CSF solute transport into basal brain parenchyma over longer times with similar distributions [140–142].

Gd injection into the cisterna magna in humans is not a practical technique; Gd injections into the lumbar CSF and tracking through the intracranial CSF have been done rarely and only when diagnosing pathological conditions. More clinically applicable although less sensitive techniques to track PVS function include diffusion imaging, PC-MRI and ultra-fast MR imaging. In rodents, T2-w diffusion techniques showed CSF in PVS preferentially moved parallel to blood flow fluctuating with cardiac pulsation, consistent with PVS-CSF movement [143] but has yet to be applied in humans. PC-MRI shows that intracranial arterial, venous and CSF pulsatility in the main cisterns, correlate with WMH burden in SVD [144–146], depends on directional flow and is less suited to understanding water mobility within the brain. Magnetic resonance encephalography (MREG) is an emerging high temporal resolution sequence, which is thought to reveal spatial-temporal patterns driven by different cardiac, respiratory and vasomotor forces highlighting that cerebral water movement is directional and cyclic with several drivers [147]. Recent work with APQ4-deficient mice suggests multi-echo ASL may provide insight into clearance mechanisms [148]. Such methods may reveal new insights into SVD, particularly large calibre vessel pulsatility effects on spatiotemporal water movement characteristics, although preclinical validation remains key.

## Discussion

Major advances have occurred in understanding human SVD, thanks to modern MRI methods; however, large gaps in knowledge remain which could be addressed through a range of SVD models and capitalising on multiple forms of clinically relevant image contrasts available with MRI. We demonstrate the need for greater transferability and reproducibility of preclinical-clinical MRI findings. There are numerous relevant rodent models for SVD. However, as yet, the imaging approaches do not appear to be taking full advantage of the knowledge derived from characterising human disease and thus limit the translational potential of rodent models in SVD. Further studies to ascertain key features of SVD and disease progression would help focus preclinical rodent models on the most salient features, whether structural or dynamic measures (e.g. WMH and PVS burden, cerebrovascular reactivity, BBB leakage etc). Many of the imaging techniques commonly used clinically have already been applied to various rodent models, including structural MR and techniques to investigate BBB integrity. However, several structural sequences are necessary in human MRI to capture the features properly and this approach could improve the yield of preclinical MRI. Closer matching of clinical to preclinical imaging protocols may aid comparisons of data and provide a fuller picture of differences in SVD disease progression and manifestations.

Only limited use has been made of ex vivo MRI and histology in studying SVD to date, though potentially relevant protocols exist for several relevant features. Greater use of these techniques may help determine which cellular mechanisms are at work, while improving understanding of the genesis and evolution of lesions and other imaging features, such as WMH and CMBs. Availability of human tissue, particularly from intermediate disease states, remains an obstacle; however, many questions can be explored with greater use of animal histology, in vivo and PM-MRI.

For dynamic imaging methods in rodents, determining the optimal anaesthetic regimen and the effect on derived imaging metrics is a significant challenge, but would help improve comparability between preclinical and hence clinical studies. Rodent models allow direct in vivo validation which would significantly advance understanding of underlying disease mechanisms, and emerging imaging methods, including synthetic MRI [149], with the potential to quantify disease-related tissue properties in vivo. Using comparable processing and analysis methods in preclinical and clinical imaging will greatly increase translational potential [113]. Application of similar image analysis methods to animal and human studies is entirely feasible, would avoid repeating the same errors, improve translation and is encouraged [22, 54, 115].

Preclinical models have also enabled advances in understanding of microvascular dysfunction underlying SVD and methods to measure paravascular transport. While the initial approaches, based on relatively invasive techniques, are not applicable in routine clinical studies, it has stimulated interest in alternative imaging methods for humans which show some promise.

Imaging of SVD is key to advancing understanding of disease pathophysiology and aiding the development of novel treatments. There is immense untapped potential for clinical research to inform preclinical work and vice versa. To maximise the benefits of research into SVD, there is a need for greater engagement and active collaborations between clinical and preclinical researchers to develop research programmes taking full account of the latest advances in both domains.

## Electronic Supplementary Material

ESM 1 (DOC 27 kb)

## Data Availability

Supplementary material including the search strategy is available on the Translational Stroke Research website.
