# Supplementary material for: A Review of Translational Magnetic Resonance Imaging in Human and Rodent Experimental Models of Small Vessel Disease
Source: Transl Stroke Res. 2020 Sep 16;12(1):15–30. doi: 10.1007/s12975-020-00843-8 (PMC7803876; doi:10.1007/s12975-020-00843-8)
Supplement: Supplementary file 1 — (DOC 27 kb) [file 12975_2020_843_MOESM1_ESM.doc]

Supplementary information for A review of translational magnetic resonance imaging in human and rodent experimental models of small vessel disease

Search strategy terms

Database: Ovid MEDLINE(R) <1946 to April Week 2 2020>, Embase Classic+Embase <1947 to 2020 April 17>

Search Strategy:

--------------------------------------------------------------------------------

1 (rodentia or rodent or rodents or murinae or mouse or mice or mus or musculus or murine or woodmouse or apodemus or rat or rats or rattus or norvegicus or guinea pig or guinea pigs or cavia or porcellus or hamster or hamsters or mesocricetus or cricetulus or cricetus or gerbil or gerbils or jird or jirds or meriones or unguiculatus or jerboa or jerboas or jaculus or chinchilla or chinchillas or beaver or beavers or castor fiber or castor canadensis or sciuridae or squirrel or squirrels or sciurus or chipmunk or chipmunks or marmot or marmots or marmota or suslik or susliks or spermophilus or cynomys or cottonrat or cottonrats or sigmodon or vole or voles or microtus or myodes or glareolus).mp. (7792072)

2 exp cerebral small vessel disease/ (663744)

3 exp Dementia/ or exp Frontotemporal Dementia/ or exp Dementia, Vascular/ or exp Dementia, Multi-Infarct/ (525719)

4 lacun*.mp. (30317)

5 deep infarct*.mp. (317)

6 subcortical infarct*.mp. (3671)

7 deep stroke*.mp. (16)

8 silent stroke*.mp. (466)

9 SVD*.mp. (6548)

10 exp stroke/ (341983)

11 microvascular disease.mp. (4091)

12 exp mild cognitive impairment/ or MCI.mp. (71334)

13 small vessel disease.mp. (9054)

14 2 or 3 or 4 or 5 or 6 or 7 or 8 or 9 or 10 or 11 or 12 or 13 (1346297)

15 exp MRI/ (943375)

16 magnetic resonance.mp. (2016530)

17 exp DCE/ (3023)

18 DCE.mp. (13701)

19 DTI.mp. (25998)

20 diffusion tractography imaging.mp. (13)

21 dynamic* contrast enhance*.mp. (15994)

22 cerebrovascular reactivity.mp. (3261)

23 ((oxygen challenge or hypercapnia or cerebrovascular reactivity) and (BOLD or Blood oxygenation level dependen* or fMRI or MRI)).mp. (1755)

24 (MRS or Magnetic resonance spectroscopy).mp. (331440)

25 (flow imaging adj5 MR*).mp. (194)

26 (phase contrast adj5 MR*).mp. (4456)

27 translat*.mp. (715255)

28 retranslat*.mp. (284)

29 translational medical research.mp. (10808)

30 exp translational medical research/ (27507)

31 protocol.mp. (816501)

32 27 or 28 or 29 or 30 or 31 (1517944)

33 ((MRI or magnetic resonance or NMR or MRM or <mu>MRI or microMRI) and (histolog* or microscopy or postmortem)).mp. (180252)

34 chronic hypertension.mp. [mp=ti, ab, ot, nm, hw, fx, kf, ox, px, rx, ui, sy, tn, dm, mf, dv, kw, dq] (7756)

35 hypertension.mp. or exp hypertension/ [mp=ti, ab, ot, nm, hw, fx, kf, ox, px, rx, ui, sy, tn, dm, mf, dv, kw, dq] (1433922)

36 15 or 16 or 17 or 18 or 19 or 20 or 21 or 22 or 23 or 24 or 25 or 26 or 33 (2086226)

37 2 or 3 or 4 or 5 or 6 or 7 or 8 or 9 or 10 or 11 or 12 or 13 or 34 or 35 (2660454)

38 1 and 32 and 36 and 37 (849)

39 limit 38 to humans (339)

40 remove duplicates from 39 (302)
